# Validation of the BC-Brain Patient-Reported Outcome Questionnaire for Patients with Central Nervous System Tumours Treated with Radiotherapy

**DOI:** 10.3390/curroncol29040228

**Published:** 2022-04-16

**Authors:** Ling Yan, Alan Nichol, Robert Olson

**Affiliations:** 1 BC Cancer-Prince George, Prince George, BC V2M 7E9, Canada; ling.yan@alumni.ubc.ca; 2 School of Population and Public Health, University of British Columbia, Vancouver, BC V6T 1Z3, Canada; 3 BC Cancer-Vancouver, Vancouver, BC V5Z 4E6, Canada; anichol@bccancer.bc.ca; 4 Department of Surgery, University of British Columbia, Vancouver, BC V5Z 1M9, Canada

**Keywords:** quality of life, patient report outcome, brain metastases, radiotherapy, questionnaires

## Abstract

The BC-brain questionnaire was developed by BC Cancer to detect health problems in patients with central nervous system (CNS) tumours in routine clinical care, treated with radiotherapy (RT), as part of the Prospective Outcomes and Support Initiative (POSI). This study aimed to present and validate the BC-brain questionnaire in patients with brain metastases (BrM) treated with RT. The BC-brain questionnaire was constructed with three subscales: mobility, thinking and CNS symptoms. Patients with BrM from five BC Cancer centres completed this questionnaire at first visit and subsequent follow-up appointments. A total of 365 patients finished the first and 105 finished the follow-up questionnaire. Summary scores of each subscale were calculated. Mobility, thinking and subtotal score showed good reliability with Cronbach’s α > 0.7. Multitrait scaling analysis showed good convergent and divergent validity. The correlations between subscales ranged from 0.262 to 0.456 for baseline and from 0.378 to 0.597 for follow-up. Patients on dexamethasone had worse performance. Patients with a KPS of </=70 had worse performance than patients with a KPS of >70. In general, this BC-brain questionnaire has good reliability and validity, and is proper to use as an option for a patient-reported outcome (PRO) instrument to measure the quality of life in BrM patients treated with RT.

## 1. Introduction

**Figure 1:**
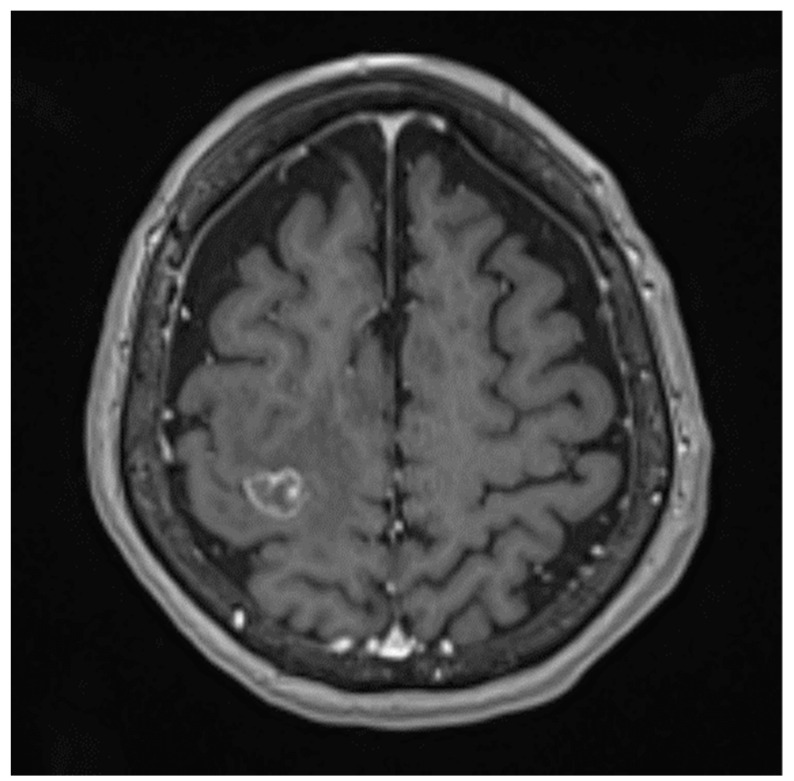
Solitary BrM on T1-weighted MRI with gadolinium.

Brain metastases (BrM) occur in approximately 20~40% of cancer patients during the course of disease [1,2,3]. The median survival is approximately 3–6 months following whole-brain RT (WBRT) [2,4], 11 months following stereotactic radiosurgery (SRS) and 2 months with supportive care only [5]. Common symptoms of BrM include headache, nausea, seizure and neurocognitive impairments, which have a major negative impact on patients’ quality of life (QoL) [6]. The treatment for patients with BrM remains an integral balance between life expectancy and patients’ QoL. Figure 1 displays a solitary BrM on MRI.

Traditionally, patients’ functional status, as measured by the Karnofsky performance score (KPS) or the Eastern Cooperative Oncology Group (ECOG) performance status, is one of the key factors to assess a patient’s fitness for treatment [7,8,9]. They are one-dimensional measures that are mostly based on the physical functions of patients; such measures are considered insufficient to describe patients’ overall QoL. A rising concept is health-related QoL (HRQOL), which was defined by the US Food and Drug Administration as “a multi-domain concept that represents the patient’s general perception of the effect of illness and treatment on physical, psychological, and social aspects of life” [10,11,12]. HRQOL was first accepted as one of the complimentary outcomes in patient care, but is increasingly being regarded as a primary outcome to guide treatment decisions.

Patient-reported outcomes (PROs) are “any information on the outcomes of health care obtained directly from patients without modification by clinicians or other health care professionals” [13,14,15,16]. A PRO is a good complement to physicians’ assessments to capture information from the patients’ perspective. QoL measures are often examples of PROs, but not all PROs are valid QoL measures, as some are not validated multi-domain questionnaires.

In brain cancer patients, the commonly used PRO instruments for HRQOL were developed for use in clinical trials, and include the functional assessment of cancer therapy–brain (FACT-Br) to be used with functional assessment of cancer therapy—general (FACT-G); the European Organization for Research and Treatment of Cancer (EORTC) (EORTCQLQ-BN20) that can be used together with EORTC core quality of life questionnaire (QLQ-C30); the MD Anderson symptom inventory for brain tumour (MDASI-BT); the brain symptom and impact questionnaire (BASIQ) and their translated versions [17,18,19,20,21]. Among them, the former two were the most popular. It has come to our attention that the pre-existing instruments were either lengthy or not designed/validated for BrM groups receiving RT or not designed for use in routine clinical care. This has driven the design of this questionnaire for patients with tumours of the central nervous system (CNS), either as primary brain tumours or BrM, which is reasonably short, yet comprehensive enough to cover brain-metastases-specific symptoms to better reflect their HRQOL. Importantly, it was designed to be practically useful in routine clinical care while seeing patients on or shortly after RT.

The objective of this work was to provide an overview of the administration of this BC-brain questionnaire at BC Cancer and examine its validity among patients receiving RT for BrM.

## 2. Materials and Methods

This BC-brain questionnaire was designed in-house at BC Cancer. BC Cancer (http://www.bccancer.bc.ca/ (accessed on 28 February 2022)), part of Provincial Health Services Authority, operates six regional cancer centres (Abbotsford, Kelowna, Prince George, Surrey, Vancouver and Victoria), providing assessment and diagnostic services, chemotherapy, radiation therapy and supportive care. Each of BC Cancer’s centres delivers cancer treatment based on provincial standards and guidelines established by BC Cancer [22].

Designed by a group of oncologists, and a nurse practitioner with extra expertise in CNS malignancies, the initial question pool consisted of the highlighted symptoms listed by this group and questions similar to those found on commonly available HRQOL questionnaires, such as the FACT and EORTC questionnaires. After item deduction, the final version consists of 24 questions. The first question is a general rating of the overall QoL with a scale from 0 to 10 (with a higher score representing better QoL) in the past three days. Questions 2 to 15 rated different aspects of QoL with a Likert scale from 0 to 4 (0 = not at all, 1 = a little bit, 2 = somewhat, 3 = quite a bit and 4 = very much.), with a higher score representing higher severity of symptoms. Question 16 to 24 were excluded from this validation because they were not QoL-related: questions 16 to 18 were specific symptoms from rare CNS issues that require specialized management, and questions 19 to 24 were designed for medicine administration (a full version of this questionnaire is attached in the appendix). The work reported in this study will focus on the 14 QoL-related questions (namely, questions 2–15), using question 1 as a reference for validation purposes.

The construct of the 14 questions was categorized into 3 subscales. The first subscale measures mobility and consists of questions 1 to 4; the second subscale measures thinking and consists of questions 5 to 8; and the third subscale measures CNS symptoms and consists of questions 9 to 14.

The first administration of the questionnaire was delivered to patients with an electronic device on their first visit. Patients could choose to answer or skip a question. Their answers were stored in the BC-brain database electronically. In the absence of an electronic device, a printed version was given to the patient and their answers were later entered into the BC-brain database by trained staff. The follow-up happened in a two-week to two-month interval. Patients who came back to the cancer centres answered the questionnaire on an electronic device, while patients who did not were followed up by phone. The minimum interval was set to avoid patients remembering their previous answer and to allow time to change, and the maximum interval was set to cover a reasonably good sample size for the follow-up. In this validation work, if a patient skipped no more than five questions, we considered that a “completed” case in this analysis.

We recruited patients from 5 of BC Cancer’s regional care centres: Abbotsford Centre, Prince George Centre, Kelowna Centre, Surrey Centre and Vancouver Centre. We included patients if they met the following criteria: aged 18 years or older, diagnosed with BrM and were seen at BC Cancer for RT. Patients were excluded if they were under 18 years of age, were diagnosed with a primary brain tumour or if they did not complete the BC-brain questionnaire.

Data linkage was performed with the cancer agency information system (CAIS) using BC Cancer ID to retrieve demographic and treatment information. Scores for subscales were linearly converted in a 0–100 scale during the analysis for ease of reporting and result interpretation, with a higher score being interpreted as more severe symptoms and thus worse HRQOL. Item 1 was converted with a lower score representing a better HRQOL on a 0–100 scale to be conceptually consistent with other items. Missing data were checked using Little’s test and imputation was performed when applicable.

Internal consistency was tested using Cronbach’s α and α ≥ 0.7 was considered statistically reliable [23,24]. Convergent and divergent validity were tested by multitrait scaling analysis [25,26,27]. Convergent validity was established when item–own scale (corrected for overlap) was greater than 0.4; divergent validity was established if the item–other scale correlations were lower than item–own scale correlation [21,28,29]. The correlations between subscales were also reviewed. It was hypothesised that all three subscales would have a relatively fair correlation with each other because they are all aspects of HRQOL while keeping themselves distinct with different focus. Their relations with the general QoL question and subtotal score (corrected for overlap) were also examined to further establish internal consistency and validity. Ranges were reported, floor effect and ceiling effect were examined, with a percent of 15 or more in the lowest scale or a percent of 15 or more in the highest scale being considered floor effect and ceiling effect, respectively [30,31].

Known-group validity was tested to examine the capability of this questionnaire to differentiate patient groups [23]. It was hypothesised that patients with higher KPS (KPS > 70) would possibly lead to a better HRQOL in general and thus have lower result scores than patients with lower KPS (KPS ≤ 70). KPS was retrospectively collected from the CAIS system within 14 days before or after the questionnaire administering date. It was also hypothesised that patients who use dexamethasone would present with worse symptoms and thus have higher scores than those who were not put on dexamethasone. Dexamethasone usage was reported by the patients using question 20 and data were collected from the BC-brain questionnaire database. A third hypothesis is that HRQOL was not related to age (age ≥ 60 vs. age < 60) or primary sites (lung vs. other sites).

Responsiveness was tested to examine the capability of this questionnaire to detect the change of HRQOL over time [23,24,32]. Patients were divided into two groups: KPS decreased (a decrease of 10 or more in KPS) group and KPS unchanged or increased (an increase of 10 or more in KPS) group. It was hypothesised that patients with improved KPS would have improved QoL and thus report lower scores to the follow-up as compared to their response at baseline. To allow change over time while keeping the loss to follow-up rate low, the interval of KPS-assessed date between baseline and follow-up was at least 1 week.

*t*-test, Spearman’s correlation, ANOVA or relevant non-parametric tests were employed when applicable for comparison. Analyses were two-tailed and a p value of less than or equal to 0.05 was considered statistically significant. Data was analysed using SPSS14.0. This research was approved by the University of British Columbia research ethical board.

## 3. Results

### 3.1. Patient Population

From July 2016 to October 2018, 419 patients were approached, and 365 patients completed the baseline questionnaire. The age of the 365 patients ranged from 23 to 88 years, with a mean of 63.3 years and standard deviation of 11.8 years. The majority were female. Patient demographic characteristics are reported in Table 1. A total of 54 patients approached did not complete the baseline assessment. Reasons for incompletion are listed in Table 2. They were older (mean age 69.4 vs. 63.3, *p* < 0.001) and had a lower KPS score than those who completed the baseline administration (65.1 vs. 78.3, *p* < 0.001).

Among the 365 patients who finished the baseline administration, 105 finished the full follow-up questionnaire in person, and 138 finished a short questionnaire over the phone (because the short questionnaire had a different construct than this BC-brain questionnaire, the 138 cases were not included as “complete follow-up” in the analysis of this validation work), and 122 did not complete the follow-up in either the long questionnaire or the short questionnaire. They had a lower KPS (74 vs. 80, *p* < 0.001) and higher scores in the general question, mobility, CNS symptoms and the subtotal (36 vs. 30, *p* = 0.01; 31 vs. 21, *p* = 0.001; 28 vs. 23, *p* = 0.002 and 26 vs. 21, *p* = 0.001 respectively).

### 3.2. Summary Scores

The summary of the subscale scores are presented in Table 3. The missing data was low for all items. The counts of missing questions per patient vary from zero questions per patient to five questions per patient and two patients skipped five questions. Question 1 had the highest missing rate; seven (2%) patients skipped this question. Mobility and CNS symptoms had higher mean score than thinking. All scores were low in their absolute value as compared to the range 0–100.

At baseline, no ceiling effects were observed among the subscales. Floor effects presented in all subscales, with the most observed in thinking. This remained true for the follow-up.

### 3.3. Reliability

Internal consistency was assessed using Cronbach’s α and results were presented in Table 3. Mobility, thinking and the subtotal showed high general reliability. During the follow-up, with CNS symptoms being an exception, the internal consistency remained satisfactory for mobility, thinking and the subtotal.

### 3.4. Validity

Item–scale correlations are listed in Table 3. Mobility and thinking showed excellent convergent validity with an item–own scale correlation greater than 0.4 (with one exception that the item “Do you feel hyper and/or agitated” had an item–own scale correlation of 0.34), and divergent validity with item–own scale correlation greater than the item–other scale correlation. CNS symptoms showed fair convergent and divergent validity.

Inter-scale correlations are reported in Table 4. Correlations for baseline are presented under the diagonal and correlations for follow-up are presented above the diagonal. Correlations among the subscales and the subtotal were corrected for overlap. Correlations were high among the three subscales, all being greater than or approaching 0.4. All subscales correlated well with the general QoL. All subscales correlated well with the subtotal.

### 3.5. Known Group Differentiation

334 patients at baseline and 101 patients at follow-up had a clear understanding of whether they were on dexamethasone or not. Comparisons of summary scores between groups are reported in Table 5.

The group on dexamethasone had higher statistically significant scores than the group not on dexamethasone on all subscales, the general question and the subtotal. It remained true on mobility, CNS symptom and subtotal during follow-up.

Patients in the low KPS group (70 and below) had higher statistically significant scores on all three subscales, the general question and the subtotal than the high KPS group (80 and above). These differences remained significant during follow-up too.

No differences were detected among the age groups or the primary site groups.

### 3.6. Responsiveness

All 105 patients met the criteria for the time interval required for KPS comparison. In total, 51 patients had decreased KPS while 54 patients had unchanged or increased KPS. For the KPS decreased group, we could observe an increase in score on the general question (40 vs. 28, *p* = 0.002), although CNS symptoms scores decreased slightly (22 vs. 27, *p* = 0.041). For the KPS unchanged or increased group, we could observe a decrease in score in CNS symptoms (18 vs. 24, *p* = 0.006) and subtotal (16 vs. 21, *p* = 0.003). No significant changes were detected on other subscales.

## 4. Discussion

This provincial study demonstrated good validity of this multi-dimensional BC-brain questionnaire developed for patients receiving radiotherapy. It was designed for use in both primary and metastatic brain tumour patients and was short in length. Specifically, it was developed for routine clinical care, as compared to many other questionnaires that were designed for clinical trials. This study was the first study to validate this questionnaire in patients with metastatic brain tumours.

We observed floor effects and no ceiling effects. This could be explained by the impression that not all cancer patients will present with severe symptoms, or their mobility/thinking functions were limitedly affected. This was also in agreement with the fact that their KPS scores in general were high. Similar observations were reported for the EORTC QLQ-BN20, where all dimensions had floor effects but no ceiling effect [33,34].

The inter-scale correlation analysis showed close correlations among the three subscales. This agreed with our hypothesis that the three subscales were non-orthogonal key aspects of HRQOL. They closely related to each other while also being able to reflect distinctive aspects of HRQOL.

Responsiveness over time was not supported when patients were stratified by their KPS changes. Several previous studies reported “lack of significant changes in scores over time” and “no significant changes in the FACT-G and FACT-Br subscales except for in the physical well-being subscale of the FACT-G” on the BrM population [3,33]. However, other reports observed increased levels of emotional distress, future uncertainty, visual disorder, motor dysfunction, seizures, drowsiness and weakness of both legs in brain cancer patients whose KPS had deteriorated [19]. Our observation on responsiveness could be a result of following factors: Certain BrM patients with rapidly deteriorating health status may be too ill to fill out questionnaires, and those who are able to complete at follow-up may have relatively high and stable HRQOL [3,17,19,35]. Additionally, the fact that palliative treatments were actively taking place between baseline and follow-up, and thus may have contributed to the declination of the summary scores [3,35].

This study should be interpreted in the context of its strengths and limitations. We had a large sample size compared to many previous studies [3,17,23,36,37,38,39]. It also met recommendations on sample size in PRO measures in the literature [40,41,42,43]. Large sample size was a direct benefit of integrating the questionnaire into the BC Cancer Prospective Outcomes and Support Initiative (POSI) system [13]. The POSI system was used across BC Cancer Agencies to collect reliable, accurate and tractable patient report outcomes, although one centre did not adopt the BC-brain questionnaire. Data was collected and managed in a systemic manner by trained staff and data grew on a daily basis. Thanks to the adoption of the EMR, demographic and treatment information was retrieved from the systems without patient involvement and thus reduced patient’s burden and avoided recall bias. One limitation of the study is the loss to follow-up. Loss to follow-up has been a long-standing issue in the area of patient-reported outcomes, which is also apparent in metastatic brain tumour populations [3,19,35]. Our results showed that patients not completing the questionnaire had a lower KPS, which may suggest that they were too unwell to take the questionnaire. In general, the population-based nature of this study is a particular strength with its patient coverage and generalizability.

## 5. Conclusions

This BC-brain questionnaire was designed to be clinically useful to guide patient care. It has good reliability and validity. It is short in length and is easy to administer without adding much patient burden in routine clinical care. It could serve as an option for a PRO instrument to measure the quality of life in BrM patients treated with radiotherapy. Future work on validation could seek to improve data collection from patients during the follow-up phase. Future research could explore the feasibility of including HRQOL in patient selection and treatment decision making for BrM patients.

## Figures and Tables

**Table 1:** Characteristics of patient population.

Characteristics	No. of Patients (Percent)
Gender	
Female	211 (58%)
Male	154 (42%)
KPS	
90–100	171 (47%)
70–80	129 (35%)
50–60	53 (15%)
<50	12 (3%)
Primary Tumour Site	
Lung	207 (57%)
Breast	64 (18%)
Melanoma	31 (9%)
Gastro-intestinal	23 (6%)
Genito-urinary	20 (5%)
Others	20 (5%)
Treatment Centre	
Abbotsford Centre	50 (14%)
Prince George Centre	26 (7%)
Surrey Centre	30 (8%)
Kelowna Centre	12 (3%)
Vancouver Centre	247 (68%)
Interpreter Used	
Yes	35 (10%)
No	330 (90%)
Follow-up	
Complete in person	105 (29%)
Complete over the phone	138 (38%)
Incomplete	122 (33%)

**Table 2:** Reason for incompletion.

Reason for Incompletion	Count (Percent)
Baseline (*n* = 54)	
Declined	27 (50%)
Unfit/unresponsive	13 (24%)
No interpreter	6 (11%)
Missed	4 (7%)
In hospital/hospice	1 (2%)
No reason	3 (6%)
Follow-up (*n* = 122)	
Unable to contact	31 (25%)
In hospital/hospice	28 (23%)
No record in system	26 (21%)
Deceased	15 (12%)
Not treated	6 (5%)
Incorrectly coded as complete	5 (4%)
No interpreter	3 (2%)
Unfit/unresponsive	2 (2%)
Declined	2 (2%)
First attempt	2 (2%)
Missed	1 (1%)
Second attempt	1 (1%)

**Table 3:** Summary scores.

Subscale	Mean(SD)	Floor No. (%)	Ceiling No.(%)	Range	Cronbach’s α	Item–Own Scale Correlation	Item–Other Scale Correlation
Baseline							
General	31.8 (20.4)	39 (11%)	1 (0%)	0–100	--	--	--
Mobility	24.4 (23.3)	202 (55%)	13 (4%)	0–100	0.85	0.56–0.73	0.29–0.44
Thinking	17.6 (17.0)	253 (69%)	2 (1%)	0–87.5	0.76	0.34–0.62	0.26–0.48
CNS Symptoms	24.8 (15.2)	142 (39%)	0 (0%)	0–75	0.63	0.25–0.48	0.15–0.57
Subtotal	22.6 (14.3)	187 (51%)	0 (0%)	0–75	0.83	--	--
Follow-up							
General	37.6 (19.9)	4 (4%)	0 (0%)	0–90	--	--	--
Mobility	22.7 (24.9)	67 (64%)	5 (5%)	0–100	0.88	0.53–0.73	0.28–0.52
Thinking	14.7 (19.3)	81 (77%)	1 (1%)	0–81.3	0.81	0.32–0.64	0.20–0.54
CNS Symptoms	19.7 (13.1)	57 (54%)	0 (0%)	0–50	0.49	0.14–0.38	0.03–0.44
Subtotal	19.2 (14.7)	69 (66%)	0 (0%)	0–57.1	0.84	--	--

**Table 4:** Inter-scale correlations.

Baseline\Follow-Up	General	Mobility	Thinking	CNS Symptoms	Subtotal
General	--	0.48	0.42	0.40	0.54
Mobility	0.46	--	0.60	0.38	0.60
Thinking	0.26	0.44	--	0.42	0.63
CNS Symptoms	0.40	0.43	0.44	--	0.44
Subtotal	0.49	0.51	0.52	0.51	--

All correlations are significant at 0.01 level (2-tailed).

**Table 5:** Known-group comparison.

		General	Mobility	Thinking	CNS Symptoms	Subtotal
		Mean (SD)	Mean (SD)	Mean (SD)	Mean (SD)	Mean (SD)
**Baseline**						
Dex	No use	28.2 (18.1)	16.8 (19.1)	14.8 (15.9)	22.0 (14.9)	18.5 (13.5)
	Use	33.7 (21.1)	29.1 (24.4)	20.0 (18.0)	27.0 (15.3)	25.6 (14.4)
	*p*	0.010	<0.001	0.006	0.003	<0.001
KPS	>70	27.9 (18.4)	18.1 (18.8)	15.6 (16.0)	22.6 (14.4)	19.3 (12.6)
	</=70	39.6 (22.0)	37.3 (26.3)	21.6 (18.3)	29.1 (16.0)	29.3 (15.2)
	*p*	<0.001	<0.001	0.001	<0.001	<0.001
**Follow-up**						
Dex	No use	35.1 (18.6)	18.5 (24.5)	11.9 (16.4)	17.3 (12.7)	16.1 (13.5)
	Use	41.7 (21.0)	28.7 (25.2)	19.7 (22.6)	22.6 (13.0)	23.9 (15.4)
	*p*	0.097	0.045	0.058	0.018	0.010
KPS	>70	31.5 (17.6)	13.2 (16.2)	9.7 (14.8)	17.3 (13.2)	13.9 (11.7)
	</=70	45.7 (20.1)	35.4 (28.7)	21.5 (22.4)	23.0 (12.5)	26.1 (15.4)
	*p*	<0.001	<0.001	0.003	0.026	<0.001

## Data Availability

The data presented in this study are available on request from the corresponding author. The data are not publicly available due to REB approval and BC Cancer Data Access Requests guidelines.
